# Model and design of real-time control system for aerial variable spray

**DOI:** 10.1371/journal.pone.0235700

**Published:** 2020-07-23

**Authors:** Yangyang Liu, Yu Ru, Liti Duan, Rongjia Qu

**Affiliations:** College of Mechanical and Electronic Engineering, Nanjing Forestry University, Nanjing, China; Nanyang Technological University, SINGAPORE

## Abstract

The dosage sprayed upon per unit area is an important index to measure the effects of pesticide application. Owing to the fact that parameters such as flight height, flight speed, and spray swath can change at any given time, it is impossible to ensure a consistent amount of pesticide application per unit area during the course of aerial variable spray. In order to ensure a consistent amount of pesticide application per unit area, a set of control models of aerial variable spray using an unmanned aerial vehicle (UAV) was proposed, and the corresponding control system was developed based on the technology of aerial variable spray. According to the change of flight parameters, this system was able to adjust the opening degree of solenoid valve through the control model of aerial variable spray. After that, the amount per unit time would change to ensure a consistent amount of pesticide application per unit area, which effectively avoided the phenomenon of uneven pesticide application and improved the accuracy. According to the actual demand for the area in need of pesticide application, the operator can manually control the amount of pesticide applied and change the dosage sprayed upon per unit area to achieve a better effect. Through field tests, it was verified that the system has high accuracy of variable control. The deviation range was between 0.11% and 9.79%, which met the demands of agricultural aviation pesticide application. Furthermore, the system had strong stability for working continuously for more than 6 h at 30°C to meet the environmental requirements of pesticide application via UAV. All the data related to the pesticide application were stored in this system, which provided a reference for the further study of the precision technology in pesticide application. The model proposed in this paper also provided a theoretical basis for the technology development of aerial variable spray.

## 1. Introduction

There are three main methods used to apply pesticides in China including manual application, mechanical application on the ground, and aviation application [1,2]. Compared with the traditional manual application and mechanical application, aviation application has the advantages of fast speed, low cost and high efficiency. What’s more, this method cannot be limited by the factors such as crop growth and geographical conditions. The utilization of the timely aviation application is especially effective in the prevention and treatment of sudden or explosive diseases, insects, and weeds [3–8]. Therefore, in order to meet the needs of different agricultural conditions and improve the accuracy of pesticide application, aerial variable spray application will be an important development direction [9–11]. The application technology of aerial variable spray can ensure that the dosage sprayed upon per unit area always remains the same, which helps to avoid the phenomenon of non-uniformity of spray when the flight parameters of aircraft changed [12]. Furthermore, aerial variable spray can also adjust the dosage sprayed upon per unit area to meet the different needs of the spray area. In this way, the pesticide waste caused by non-differential application, and insufficient amount of pesticide application will be avoided. As we all know the key technology of aerial variable spray is to monitor the pesticide application parameters and variable control. There are a lot of studies focusing on the monitoring of application parameters all over the world, however, the research on variable control is scarce.

International scholars have also made some achievements in the field of aerial variable spray control. For examples, Adapco Company developed the Wingman GX product [13], Xing et al. developed the ground monitoring system of variable spraying for UAV of plant protection [14], and Wang et al. developed the variable system for small UAV [15]. The application flow can be manually controlled by the above aerial variable spray system. The ground monitoring system designed by Zhang et al. has the ability to change the flight state by remote-distance control, and is able to change the amount of pesticide spray per unit area after that [16]. However, it should be noted that this experience-based approach to man-made regulation of spray amount makes it difficult to ensure spray precision [17]. The control system of variable spray designed by Zhang et al. [12], as well as the variable-rate spray system designed by Lian et al. [18]or Yao et al. [19], can automatically adjust the amount of pesticide application in the pipeline according to the change of flight speed of the aircraft. The variable-rate aerial application system developed by Stevenj et al. can implement variable-rate control according to the areas under the flowrate-time curves [20]. The variable spray system for UAV was developed by Wen et al. can adjust the flow rapidly according to the prescription value set in the prescription map [21]. As for the variable-rate fungicide application designed by Tackenberg et al. [22,23], as well as the real-time variable-rate herbicide application designed by Dammer et al. [24], they are based on the image monitoring technology of sensors to change the local application dose. The variable spraying system of plant protection UAV designed by Zhang [25], and the variable rate application system for sprayers designed by Han et al. own the ability to control the amount according to crop growth, disease, and insect pests [26]. However, the methods for controlling the amount of pesticide within their study are limited. In their studies, they predominantly focused on remote-distance control and manual control, where the automatic control method of the amount can only be regulated according to a single variable, and it is unable to simultaneously adjust and control the amount of spray based on multiple parameters (such as the flight height, flight speed, and spray swath).

The topography is relatively complex in China, and the existing techniques and equipment in agricultural aviation cannot fully meet the demands of Chinese market [27]. International aerial variable spray equipment has not been fully used in practice of China. The main reasons for this phenomenon are that the equipment is too expensive and very difficult to be used for secondary software development, which make it unable to meet the demand in domestic aerial variable spray. There is still a lack of research on the control theory of aerial variable spray in agricultural around the world, and a complete theoretical model of aerial variable spray control is highly desirable.

Therefore, on the basis of previous research on aerial variable spray monitoring system and its related techniques, our study put forward a set of aerial variable spray models for UAV application technique as the theoretical control basis according to the current situation in China. Additionally, a real-time control system for aerial variable spray was developed, by which variable spray according to the parameters set and real-time monitoring were realized. Compared with the variable spray control system of helicopter and variable rate spray system [12,18], this system can not only automatically adjust the flow when the flight speed changes. It also can automatically adjusted flow when the flight height, spray swath and unit area of the need for dosage changes. Compared with manual control, the system is simple to operate and can accurate control flow to reduce errors caused by subjective factors. After that, the accuracy of variable control was tested and analyzed.

## 2. Theory

### 2.1 Analysis of influencing factors

In order to achieve the effective control effect of agro-forestry diseases and insect pests, the per unit area needs to meet a certain dosage of spray [28], that is, to achieve a certain spray concentration. If the spray dosage is too low, it is difficult to protect the plants from factors such as diseases, weeds, and pests. If the concentration is too high, pesticide waste and environmental pollution will occur [29–30]. In order to ensure that the spray dossge is within the optimal range and the phenomena of local less spray, leakage spray, and multiple spray are avoided, it is necessary to analyze and control the factors that may cause the uneven application of the pesticide. According to the practical experience of aerial spray of UAV, it can be observed that the nozzle and the variation of spray swath of different sprinklers can directly affect spray concentration. However, the existing UAV nozzles predominantly use centrifugal sprinklers. In this paper, the electric centrifugal nozzle is analyzed. The voltage of the nozzle directly determines the rotate speed of the nozzle, and then impacts the spray swath. In addition, the flight height, flight speed, and flow amount of the aircraft will affect the consistency of the spray concentration.

### 2.2 Establishment of mathematical model

In order to ensure the consistency of the spray concentration, it is necessary to determine the mathematical relationship between the four individual factors of flight height, flight speed, nozzle voltage as well as flow amount and the spray concentration.

#### 2.2.1 Relationship between spray volume and spray concentration

The spray concentration is the dosage sprayed upon per unit area, which is calculated from the ratio between the total spray volume and the area of spray, as shown in Formula (1):

$$C=\frac{L}{S_{a}}$$ (1)

where *L* denotes the spray volume (Unit: mL or cm^3^,1*mm*^3^ = 1*ml*); *C* denotes the spray concentration (Unit: ml·acre^-1^); and *S*_a_ denotes the area of spray (Unit: acre).

The calculation of the spray volume is presented in Formula (2) as follows:

$$L=S_{v}V_{p}t$$ (2)

where *S*_*v*_ denotes the section of valve outlet (Unit: mm^2^), *V*_p_ denotes the flow velocity of the liquid (derived from sensor) (Unit: m·s^-1^); and *t* denotes the time of spray (Unit: s).

According to Formula (2), the opening size of the solenoid valve, the flow velocity of the liquid and the time of spray determines the spray volume. Assuming that the flow velocity and the time of spray are constant, the larger the opening-size of the valve, the greater the spray volume. Since the valves are ball-shaped solenoids, the opening area can be regarded as two equal-sized circles coinciding, intersecting, or in tangent, and the valve angle is in the range of 0°-90°. When the valve angle is 0°, the two circles coincide and the valve is in the state of full open. If the valve angle is more than 0° but less than 90°, the two circles intersect. The two circles are in tangent and the valve is closed when the valve angle is 90°.

**Fig 1:**
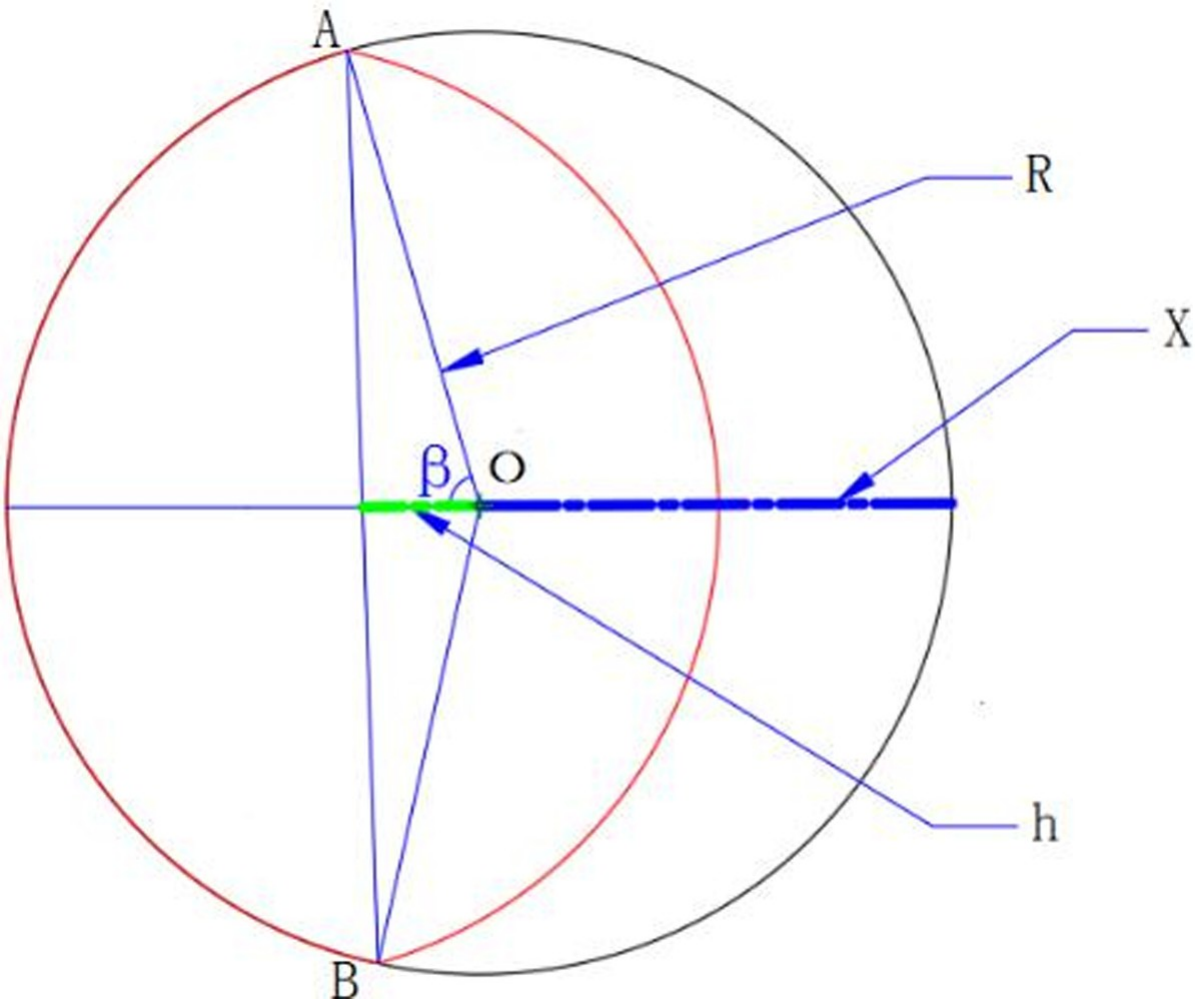


The section of valve outlet when the valve angle is between 0° and 90° is presented below in Fig 1.

As can be seen from Fig 1, the area in the red ellipse is the cross-sectional area of the valve outlet. Accordingly, the following formula can be derived:

$$\{\frac{x}{2R}=\frac{\alpha }{90{}^{\circ}}$$ (3)

2(R−h)+x=2R (4)

$$\cos\;\beta =\frac{h}{R}$$ (5)

where *R* denotes the radius of the spherical solenoid valve channel (Unit: mm); *x* denotes the maximum displacement projection of the spherical solenoid valve edge (Unit: mm); *h* denotes the distance from the middle point of the intersecting line of two circles to the center of the section of the outlet (Unit: mm); *β* denotes the half angle of the sector of the two circles intersecting (Unit: °); and *α* denotes the angle of the solenoid valve, the angle range from 0° to 90°.

The cross-sectional area of the valve outlet is added with two red semicircular areas, and the following Formulas (6) can be obtained by being combined with Formulas (3) and (4):

$$S_{v}=2(\frac{2\beta \pi R^{2}}{360{}^{\circ}}-\frac{\alpha }{180{}^{\circ}}R^{2}\sqrt{1-{\left(\frac{\alpha }{180{}^{\circ}}\right)}^{2}})$$ (6)

Combined with Formulas (5) and (6), the cross-sectional area of the valve outlet can be determined in Formula (7):

$$S_{v}=\left[\frac{\pi \arccos(\frac{\alpha }{180{}^{\circ}})}{90{}^{\circ}}-\frac{\alpha }{90{}^{\circ}}\sqrt{1-{\left(\frac{\alpha }{180{}^{\circ}}\right)}^{2}})\right]R^{2}$$ (7)

In combination with Formulas (1), (2), and (7), the relationship between the opening angle of valve and the spray concentration can be deduced, as shown in Formula (8):

$$C=\frac{\left[\frac{\pi \arccos(\frac{\alpha }{180{}^{\circ}})}{90{}^{\circ}}-\frac{\alpha }{90{}^{\circ}}\sqrt{1-{\left(\frac{\alpha }{180{}^{\circ}}\right)}^{2}})\right]R^{2}V_{p}t}{S_{a}}$$ (8)

According to Formula (8), there is a transcendental function relationship between the spray concentration and the angle of valve when the conditions of remain constant area of spray, flow velocity of the pesticide liquid, cross-section of delivery tube of pesticide, and time of spray are met.

#### 2.2.2 Relationship between flight speed and spray concentration

According to Formula (8), the area of spray is inversely proportional to the spray concentration, but the area of spray is affected by the flight speed, spray swath, and the time of spray. As such, Formula (9) can be derived from the correlation formula of particle motion as follows:

$$S_{a}=V_{f}tY$$ (9)

where *Y* denotes spray swath (Unit: m); and *V*_*f*_ denotes flight speed (Unit: m·s^-1^).

In combination with Formulas (8) and (9), the relationship between the flight speed and the spray concentration can be calculated as follows in Formula (10):

$$C=\frac{\left[\frac{\pi \arccos(\frac{\alpha }{180{}^{\circ}})}{90{}^{\circ}}-\frac{\alpha }{90{}^{\circ}}\sqrt{1-{\left(\frac{\alpha }{180{}^{\circ}}\right)}^{2}})\right]R^{2}V_{p}t}{V_{f}tY}$$ (10)

According to Formula (10), there is an inverse proportional function relation between the spray concentration and the flight speed when remain constant condition of the spray swath, flow velocity of the pesticide liquid, the cross-section of the delivery tube of pesticide, and the time of spray is achieved. And it can be concluded that the spray concentration is independent of the spray time without repeated spray. Since the variables in common formula (16) are all instantaneous variables, the spray concentration is the instantaneous spray concentration.

#### 2.2.3 Relationship between flight height and spray concentration

Spray swath is mainly affected by the flight height, so the relationship between the flight height and the spray swath should be determined first. Various forces affect the movement process of fog droplets [31]. However, the trajectory of fog droplets is not studied in depth within this paper. Therefore, some smaller forces can be ignored and only gravity will be analyzed.

**Fig 2:**
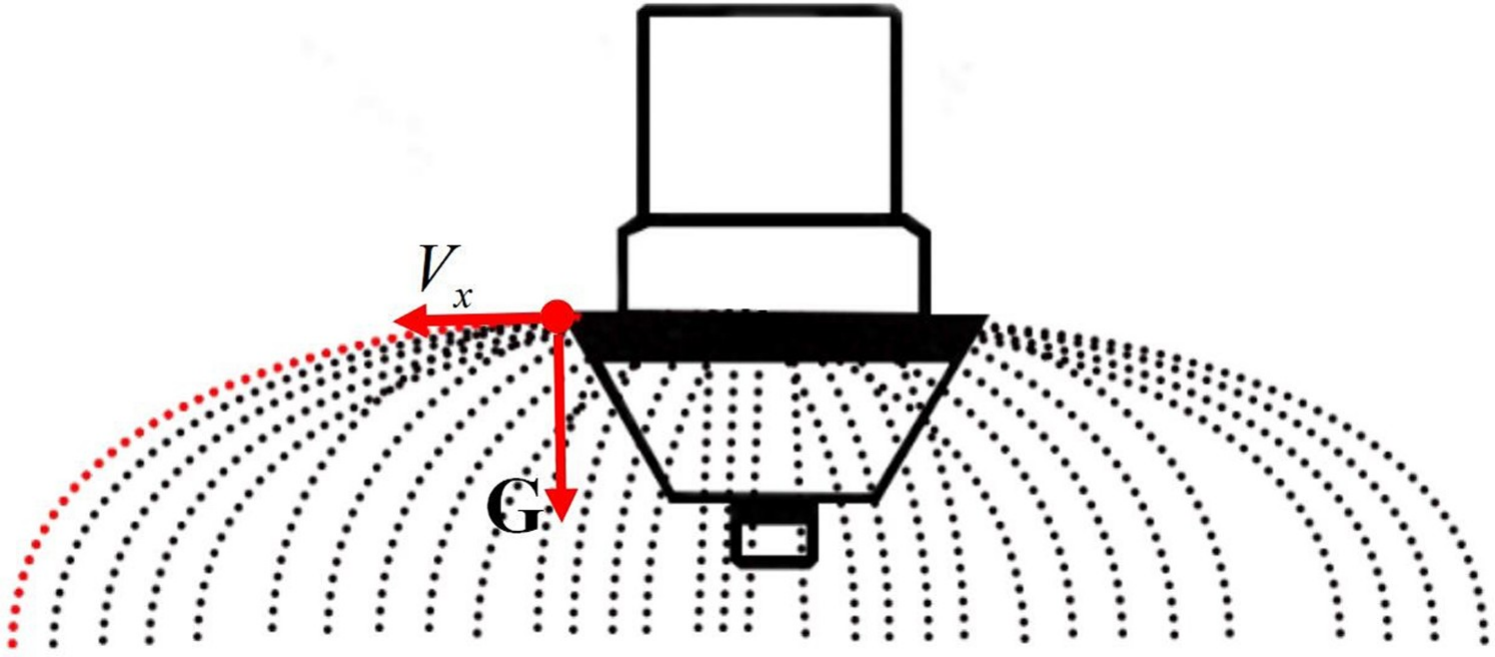


The relationship between flight height and spray swath can be derived from the relevant formula, such as Formula (11), as follows:

$$Y=V_{x}\sqrt{\frac{2H}{g}}$$ (11)

where *H* denotes the flight height (Unit: m);g denotes the gravity acceleration (Unit: m·s^-2^); and *V*_*x*_ denotes the horizontal velocity of the pesticide liquid outlet (Unit: mL·s^-1^). The schematic diagram as shown in Fig 2.

In combination with Formulas (10) and (11), the relationship between the flight speed of UAV and the spray concentration is presented as Formula (12):

$$C=\frac{\left[\frac{\pi \arccos(\frac{\alpha }{180{}^{\circ}})}{90{}^{\circ}}-\frac{\alpha }{90{}^{\circ}}\sqrt{1-{\left(\frac{\alpha }{180{}^{\circ}}\right)}^{2}})\right]R^{2}V_{p}}{V_{f}V_{x}\sqrt{\frac{2H}{g}}}$$ (12)

According to Formula (12), the relationship between the flight height and the spray concentration is power function under the remain constant condition of the spray swath, flow velocity of the pesticide liquid, the cross-section of the delivery tube of pesticide, flight speed, and the horizontal velocity of the pesticide liquid outlet.

#### 2.2.4 Relationship between nozzle voltage and spray concentration

In this paper, Auz900 of the electric centrifugal nozzle is selected. There is no direct formula for calculating the relationship between the nozzle voltage and the spray swath. Formula (13) is obtained by means of sine curve approximation on the basis of the previous experimental data of the research group [32–34]. The following formula can be given:

$$Y=P_{1}\sin(P_{2}U+P_{3})$$ (13)

Where *U* denotes the voltage of nozzle motor (Unit: V); *P*_1_,*P*_2_, and *P*_3_ are regarded as the constant, *P*_1_ = 19.97, *P*_2_ = 0.006539, and *P*_3_ = 2.961.

The trigonometric function relation between the nozzle voltage and the spray swath is obtained according to Formula (13).

The preliminary tests of the research group are all the spray swath data obtained by changing the nozzle voltage when the altitude is 4 m. Therefore, the relationship between the horizontal velocity of the liquid outlet (*V*_*x*_) and the nozzle voltage (U) can be calculated. Formula (14) can be obtained by combining Formula (13) with Formula (11):

$$V_{x}=1.1079P_{1}\sin(P_{2}U+P_{3})$$ (14)

In combination with Formulas (12) and (14), the relationship between nozzle voltage and spray concentration can be obtained, as shown in Formula (15):

$$C=\frac{\left[\frac{\pi \arccos(\frac{\alpha }{180{}^{\circ}})}{90{}^{\circ}}-\frac{\alpha }{90{}^{\circ}}\sqrt{1-{\left(\frac{\alpha }{180{}^{\circ}}\right)}^{2}})\right]R^{2}V_{p}}{1.1079P_{1}\sin(P_{2}U+P_{3})V_{f}\sqrt{\frac{2H}{g}}}$$ (15)

According to Formula (15), the function relationship between the flight height, flight speed, nozzle voltage (*U*), angle of solenoid valve (*α*), and the spray concentration is obtained.

According to the previous research results [35–39], as well as the operation experience from several plant protection companies, the relative flight height and flight speed of UAV should be controlled within 1–5 m and 3–6 m·s^-1^, respectively. The spray concentration should be controlled in the range of 3000–6000 mL·acre^-1^, the spray volume should be controlled in the range of 20–35 mL·s^-1^, and the working voltage of the nozzle should be controlled in the range of 5–12 V, which is regarded as the optimal effect of spray. However, in order to ensure the efficiency of spray, the variation range of valve angle should be controlled between 0° and 30°. Therefore, the compensation value should be added to the mathematical model Formula (16). The mathematical model of aerial variable spray can be obtained as follows:

$$C=\frac{\left\{0.4712+4.2604\left[\frac{\pi \arccos(\frac{\alpha }{180{}^{\circ}})}{90{}^{\circ}}-\frac{\alpha }{90{}^{\circ}}\sqrt{1-{\left(\frac{\alpha }{180{}^{\circ}}\right)}^{2}}-2.478\right]\right\}R^{2}V_{p}}{1.1079P_{1}\sin(P_{2}U+P_{3})\sqrt{\frac{2H}{g}}V_{f}}$$ (16)

According to the mathematical model, the solenoid valve angle will be automatically calculated when the spray concentration (*C*), flight height (*H*), flight speed (*V*_*f*_), and nozzle voltage of Auz900 (*U*) are set. The spray volume can be adjusted by changing the opening-size of the valve. Therefore, when any of the parameters or multiple parameters change at the same time, the system can automatically calculate the corresponding valve opening-size. By changing the valve opening-size and then changing the spray volume to ensure that the spray concentration is not changed. The spray concentration obtained by the model is the instantaneous spray concentration, and the uniformity of the spray volume in the region can be ensured by ensuring the consistency of the instantaneous spray concentration.

## 3. Accuracy test of control system

### 3.1 System design

#### 3.1.1 Hardware design

**Fig 3:**
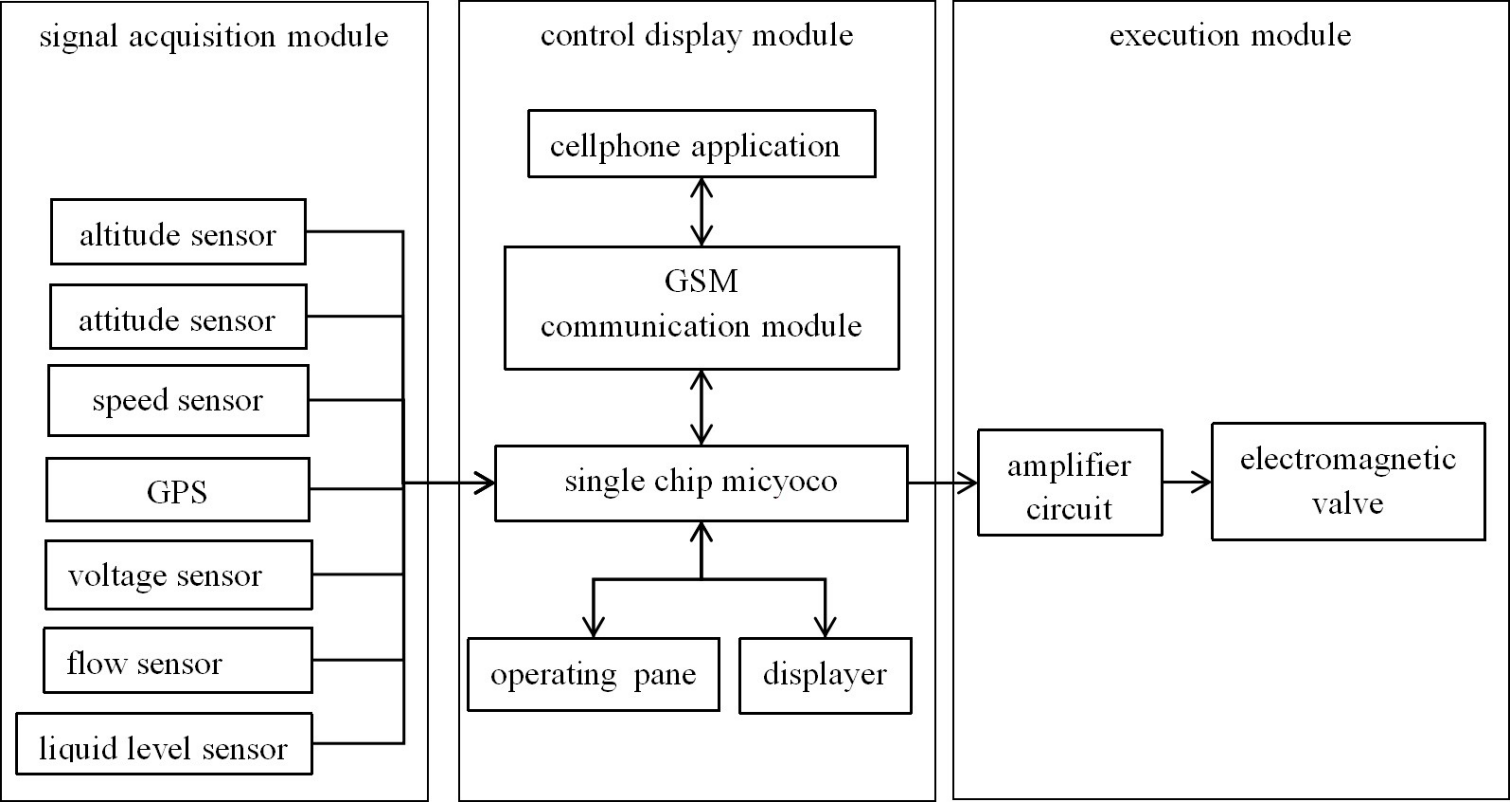


The hardware control system module of spray shown in Fig 3 mainly includes a signal acquisition module, a control display module, and an execution module. The signal acquisition module in the diagram measures the flight status signal and the status signal of spray. The altitude sensor is used to detect the flight height. The pitot tube is used to monitor the flight speed. Additionally, the GPS is for detecting the flight location, and the flight speed can be calculated according to the change of latitude and longitude within per unit time. In this paper, the flight speed obtained by calculating the coordinate change in unit time is used, and the speed monitored by pitot tube is adopted when GPS signal is not available. The gyroscope is used to detect the flight attitude while GPS is for detecting the flight location. The spray volume is detected by the turbine flow sensor, and the liquid level sensor is used to measure the level of the liquid in the spray box. Voltage sensor is for detecting the nozzle voltage.

The control display module fuses the multiple signals collected by the sensor and the input spray parameters, and calculates the actual flight state and the pesticide application. The flight status and the pesticide application could be intuitively observed in a numerical form in the display screen and on the APP of mobile phone. A single chip microcomputer of STM32F103VCT6 type was chosen as the micro-controller for the information processing and control system. The input of the microcontroller is connected with the signal acquisition module and the operating board, and the output is connected with the display and the execution module. The micro-controller outputs the control signal through the algorithm of aerial variable spray model by transforming the sensor signal, then controlling the rudder of solenoid valve by the pulse width modulation method, and finally adjusting the opening-size of solenoid valve to achieve variable spray.

#### 3.1.2 Software design

The software design of the real-time control system of aerial variable spray mainly includes five portions of data acquisition, data processing, data display, parameter setting, and wireless transmission. Among them, the data processing is the comprehensive processing procedure of all the signals. In other words, if the data collected from the information collection module is within the normal range of pesticide application, the angle of the solenoid valve will be adjusted by the single chip microcomputer based on the mathematical model of aerial variable spray. However, if one of the parameters of flight height, flight speed, nozzle voltage, or spray concentration is equal to 0, the angle of control valve will be 90° and the valve will be in the closed state. If any three items from flight height, flight speed, nozzle voltage, or spray concentration is equal to or more than the maximum of pesticide application, the angle of control valve will be 0° and the valve will be in full open state. Keil software was chosen in this study to conduct the program development to the single-chip computer.

**Fig 4:**
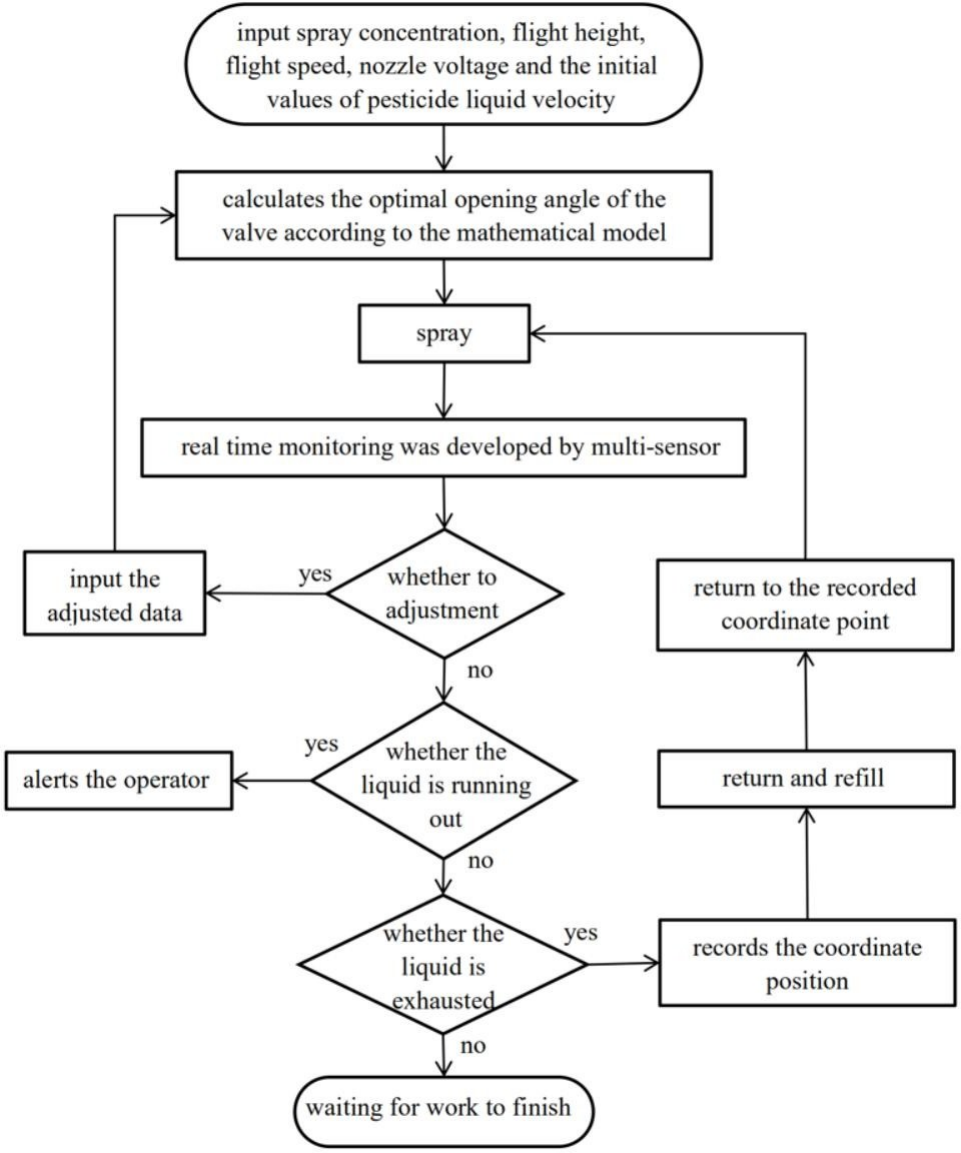


The control scheme realizes the variable spray according to the parameters set and real-time monitoring. The operator is also able to artificially control the flight status and the pesticide application to achieve the real-time monitoring. The corresponding flowchart is shown in Fig 4.

Firstly, the spray concentration, flight height, flight speed, nozzle voltage, and the initial values of pesticide liquid velocity (determined by liquid pump) should be input into the system. After that, the system automatically calculates the optimal opening angle of the valve based on the mathematical model. The flow sensor is used to measure the pesticide liquid velocity in real-time monitoring, and the opening angle of the valve according to the actual flow velocity of the pesticide liquid can be accurately calculated the system. For the spraying process, pesticide application of real-time monitoring was developed by multi-sensor. If the operator judges the need to adjust the flight parameters according to the actual situation, the flight parameters will be re-inputted, and the valve angle according to the newly inputted flight parameters will be recalculated by the system. If the liquid level sensor detects that the liquid is about to be depleted, it will alert the operator and record the coordinate position point when the pesticide liquid is exhausted. Then, it will return to the point after the liquid is replenished.

### 3.2 Testing materials and conditions

The UAV model used in this study is the "flying spider," which has four-rotor electric UAV produced by Linyi Fengyun Aeronautical Technology Company. The hydraulic pump is a brushless water pump produced by Dajiang, and the flow velocity of the pesticide liquid is controlled at 9.55 cm·s^-1^. The nozzle is an electric centrifugal nozzle of Auz900 type [34]. The test site is a farmland with a size of 0.33 acre. The test time is from July 1st to July 3rd, 2018. The average temperature is 31°C, the relative humidity of air is approximately 60%, and the wind speed is 1–3 m·s^-1^.

### 3.3 Testing purpose

The purpose is to test if the system can accurately adjust the spray volume according to the mathematical model of aerial variable spray when the flight parameters change, and further ensure the stable spray concentration. When the deviation between the actual spray concentration and the set spray concentration is less than 10%, it can be considered that the demands of agricultural aerial spray has been met [12]. Since that most of pesticide spray operations of UAV are conducted in the summer, the system needs to meet the requirements of long-term work under high temperature conditions.

### 3.4 Testing scheme

The test consists of four groups of one-way test, one group of multivariate test and a set of response time tests. In the one-way test, one of the four variables (flight speed, flight height, nozzle voltage, and spray concentration) is the variable factor, whereas the other factors remain unchanged, as shown in Table 1. Among them, G1 group is the three-level test of flight speed while keeping the flight height, nozzle voltage, and spray concentration unchanged; G2 group is the three-level test of flight height while keeping flight speed, nozzle voltage, and spray concentration unchanged; G3 group is the three-level test of nozzle voltage while keeping flight speed, flight height, and spray concentration unchanged; and G4 group is the three-level test of spray concentration while keeping flight speed, flight height, and nozzle voltage unchanged. Among them, the test group is the system control spray volume, and the control group is the manual control spray volume. Multivariate test is based on four factors and three levels of orthogonal test, which takes flight speed, flight height, nozzle voltage, and spray concentration as variable factors, as shown in Table 2. Each level test was repeated three times, and the average value was taken as the effective value of the actual spray concentration. These values were then compared with the set spray concentration, and the relative deviation was calculated.

**Table 1:** The results of the one-way tests of four groups.

					Test Group		Control Group	
Group	Flight Speed (m·s^-1^)	Flight Height (m)	Nozzle Voltage (v)	Set Spray Concentration (mL·acre^-1^)	Actual Spray Concentration(mL·acre^-1^)	Relative Deviation of Spray Concentration(%)	Actual Spray Concentration(mL·acre^-1^)	Relative Deviation of Spray Concentration(%)
	3				5252.06	9.42	5528.94	15.19
G1	5	4	8	4800	4839.55	0.82	5703.72	18.83
	6				4596.12	4.25	3783.48	21.18
		2			4677.42	2.55	6393.54	33.20
G2	4	3	8	4800	5068.73	5.60	5237.46	9.11
		5			4986.36	3.88	6829.20	42.28
			5		5269.79	9.79	4298.64	10.45
G3	4	4	6.5	4800	4903.39	2.15	5302.5	10.47
			10		5082.12	5.88	3516.84	26.73
				3000	3219.90	7.33	5173.62	72.45
G4	4	4	8	3900	4243.38	8.80	4263.90	9.33
				6000	5993.64	0.11	7272.18	21.20

**Table 2:** The results of the multivariate tests.

Group	Flight Speed (m·s^-1^)	Flight Height (m)	Nozzle Voltage (v)	Set Spray Concentration (mL·acre^-1^)	Actual Spray Concentration (mL·acre^-1^)	Relative Deviation of Spray Concentration (%)
T1	3	2	5	3600	3889.97	8.05
T2	3	3	6.5	4200	4359.06	3.79
T3	3	5	10	6000	5952.36	0.79
T4	5	2	6.5	6000	6363.48	6.06
T5	5	3	10	3600	3724.21	3.45
T6	5	5	5	4200	4096.85	2.46
T7	6	2	10	4200	4605.00	9.64
T8	6	3	5	6000	5861.03	2.32
T9	6	5	6.5	3600	3813.48	5.93

The purpose of response time test is to verify whether the system can control the spray volume in real time. Because the flight height and flight speed cannot jump and the acceleration of the uav is low, so that the response delay caused by the change of flight height and flight speed can be ignored. Therefore, it is only necessary to take voltage and spray concentration as variable parameters to conduct four-level tests respectively, as shown in Table 3. The initial value of the variable parameters are set as follows: flight speed is 4 m·s^-1^, flight height is 4 m, nozzle voltage is 0 V, spray concentration is 0 mL· acre^-1^. The monitoring system reads the variable parameters every 200ms, so the system response time calculation formula is as follows formula (17):

$$T=t_{0}+t_{1}$$ (17)

Where, *T*- system response time, s; *t*_0_-system read interval time, s; *t*_1_- valve response time, s.

**Fig 5:**
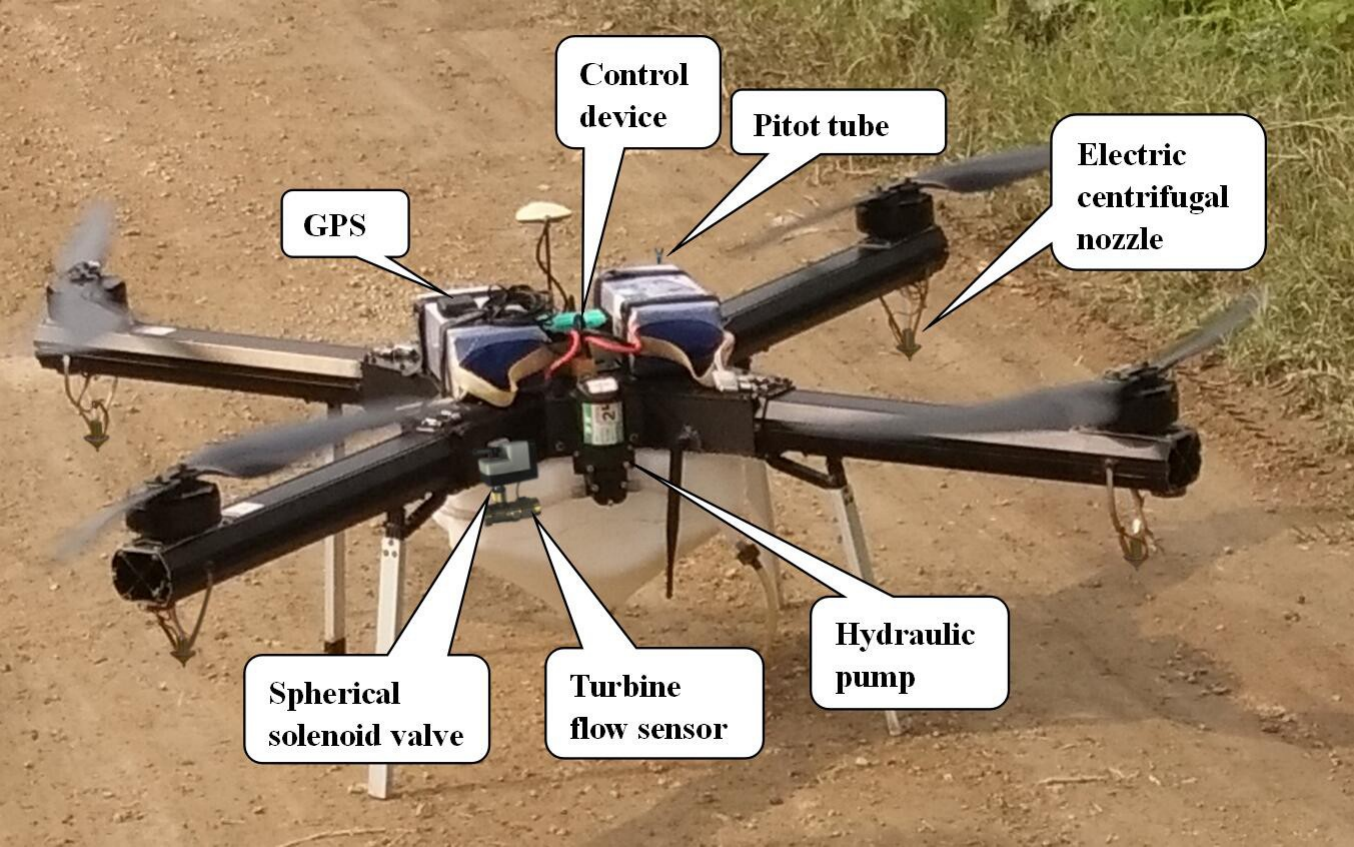


During the test, the device is mounted on the UAV and fixed in the middle of the batteries. As shown in Fig 5, the altitude sensor and GPS calibration are conducted. According to the experience of spray operation, the optimum parameters of spraying are set as follows: flight speed is 4 m·s^-1^, spray volume per unit area is 4800 mL acre^-1^, flight height is 4 m, nozzle voltage is 8 V, and the flow velocity of the pesticide liquid is 9.55 cm·s^-1^. The single factor and multi-factor tests were then carried out on 0.33 acre test site according to the planning route, and the total volume was recorded after each pesticide application. The actual spray concentration can be obtained by comparing the total volume of spray with the area of the test site, and the relative deviation of the concentration can then be obtained by comparing the actual spray concentration with the set spray concentration.

**Table 3:** The results of the system response time tests.

groups	Flight Speed(m·s^-1^)	Flight Height (m)	Nozzle Voltage (v)	The Set Spray Concentration (mL·acre^-1^)	The Corresponding Time (s)	The Corresponding Distance (m)
A0	4	4	0	0	/	/
A1	4	4	10	500	0.37	1.48
A2			8	500	0.26	1.04
A3			6.5	500	0.25	1.00
A4			5	500	0.25	1.00
A5	4	4	5	650	0.40	1.60
A6			5	800	0.27	1.08
A7			5	1000	0.47	1.88

## 4. Results and analysis

(1) The results of the one-way tests of four groups are shown in Table 1.

**Fig 6:**
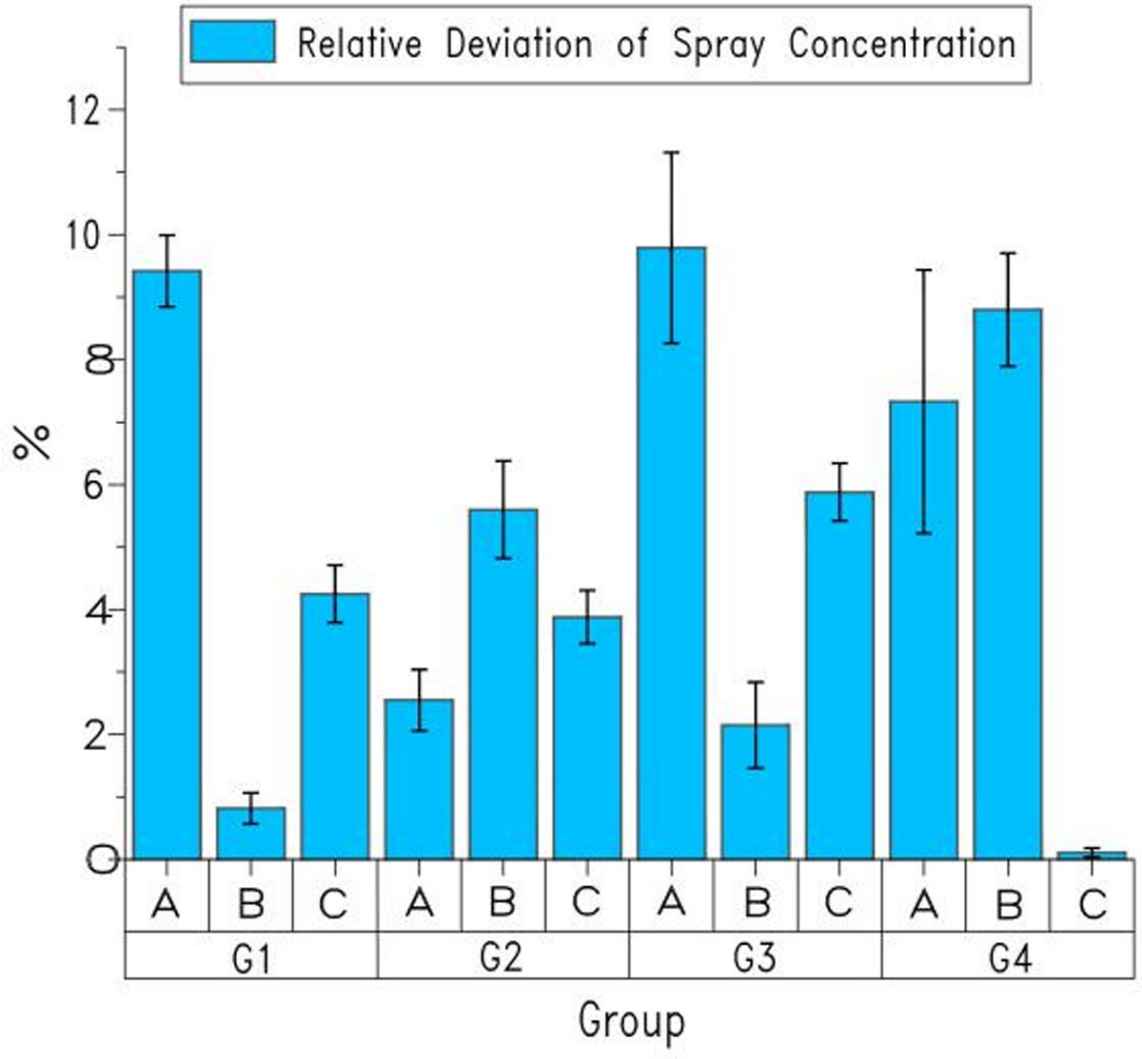


As shown in Table 1, the control system of the aerial variable could adjust the spray volume according to the changed single flight parameters to ensure the consistency of spray concentration when flight height, flight speed, spray concentration, and nozzle voltage individually changed. What’s more, the deviation between the actual spray concentration and the set value lied in the range of 0.11%-9.79%, among which the biggest deviation was 9.79%. This value was less than 10%, which showed that the actual value of the spray concentration was close to the set value when a single factor was adjusted. However, the deviation range of manual control was between 9.11% and 72.45%. Due to the large deviation, the manual control will cause the spray concentration in the spray area to be seriously uneven and the effect is poor. And from the error bar in Fig 6, it can be concluded that the degree of data dispersion was low, which indicated that the accuracy of monitoring values is high.

The one-way tests of four groups experimental results showed that the system accurately calculated the spray volume when any flight parameters changed according to the variation of parameters based on the mathematical model of aerial variable spray. The results ensured the consistency of spray concentration in farmland, and the control precision of the variable spray system was able to meet the demand of agricultural aerial variable spray.

(2) The results of the multivariate tests are shown in Table 2.

**Fig 7:**
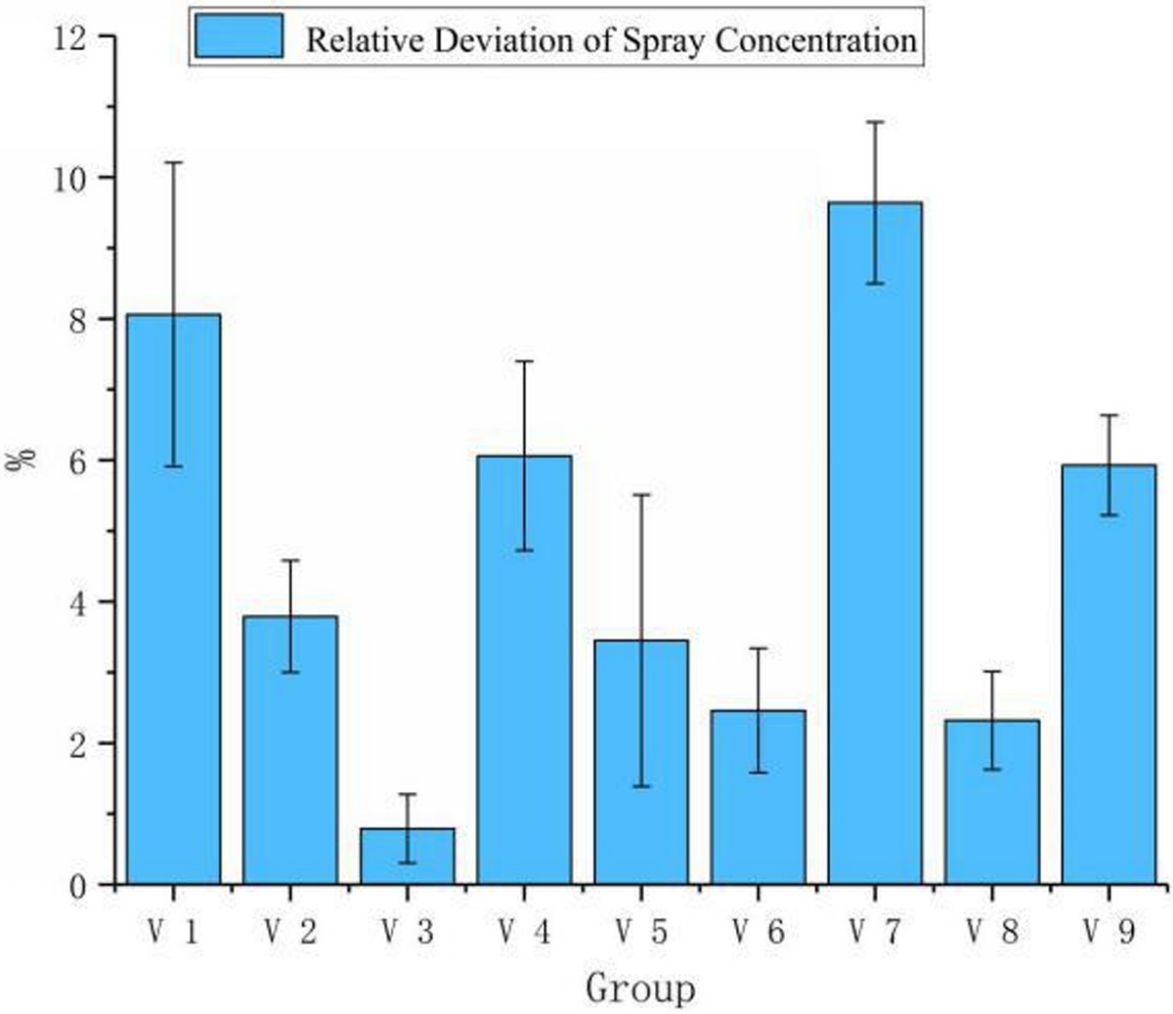


When the four parameters of flight height, flight speed, spray concentration, and nozzle voltage were simultaneously changed in the setting range, the system could determine the angle of solenoid valve through the aerial variable spray model based on Formula (16), adjusting the spray volume and obtaining the actual spray concentration. As shown in Table 2, the deviation between the actual spray concentration and the set spray concentration lies in the range of 0.79%-9.46%. The maximum deviation is 9.46%, which is less than 10%. The actual spray concentration is close to the set spray concentration, when multiple flight parameters are adjusted. And from the error bar exhibited in Fig 7, the conclusion that the degree of data dispersion is low can be drawn, which indicates that the accuracy of monitoring values is high.

The multivariate tests results showed that the system accurately calculated the spray volume when multiple flight parameters changed at the same time according to the variation of parameters based on the aerial variable spray mathematical model. The performance ensured the consistency of spray concentration in farmland, and the control precision of the variable spray system could meet the demand of agricultural aerial variable spray.

(3) The results of the system response time tests are shown in Table 3.

As shown in Table 3, the response time of the control system lied in the range of 0.25–0.47s, and the corresponding distance was between 1–1.88m, among which the biggest distance was 1.88m. At present, the positioning error of uav in the market is within 2m, so the 1.08m response distance can meet the variable spray demand of uav.

## 5. Conclusion

1. A mathematical model of the real-time control of aerial variable spray was proposed with the focus on UAV spray operation. The model established the relationship among flight speed, flight height, spray concentration, nozzle voltage, and spray volume, which provided theoretical support for the research of aerial variable spray.
2. A real-time control system of aerial variable spray was designed, and the hardware and software structures of the system were elaborated in this study. Combined with the mathematical model of aerial variable spray, the scheme of the real-time control system of aerial variable spray was put forward. The experimental results showed that when any flight parameters changed or multiple flight parameters changed at the same time, the system could accurately calculate the spray volume according to the variation of parameters based on the mathematical model of aerial variable spray. The performance ensured the consistency of spray concentration in farmland. The maximum deviation in the experimental data was 9.79% less than 10%, which met the control precision demand of agricultural aerial variable spray. The system has strong stability to work for more than 6 h at a high temperature of 31°C, which could meet the environmental requirements of aerial variable spray.
3. The system effectively solved the problem of uneven spray characteristics caused by the change of flight parameters in the process of aerial variable spray. It can also adjust the real-time spray concentration to meet the different needs of the spray areas, and the waste of pesticides and the issue of insufficient pesticides caused by indiscriminately spray will be avoided, The system improved the accuracy of spray and provided a foundation for precision spray. This study also reduced the steps of manual regulation and the control of spray volume in the process of pesticide application, which ultimately improved the efficiency.

## Supporting information

S1 File Minimal data set. (DOCX)
