# Supplementary material for: Model and design of real-time control system for aerial variable spray
Source: PLoS One. 2020 Jul 23;15(7):e0235700. doi: 10.1371/journal.pone.0235700 (PMC7377416; doi:10.1371/journal.pone.0235700)
Supplement: S1 File — (DOCX) [file pone.0235700.s001.docx]

# Minimal Data Set

**Table 1**: The data of the one-way tests, the multivariate tests and the manual control tests.

| **[Group](D:/%E5%AE%89%E8%A3%85%E8%BD%AF%E4%BB%B6/Dict/8.1.2.0/resultui/html/index.html#/javascript:;)** | | **[Flight](D:/%E5%AE%89%E8%A3%85%E8%BD%AF%E4%BB%B6/Dict/8.1.2.0/resultui/html/index.html#/javascript:;)**  **[Speed](D:/%E5%AE%89%E8%A3%85%E8%BD%AF%E4%BB%B6/Dict/8.1.2.0/resultui/html/index.html#/javascript:;)**  **(m·s^-1^)** | **[Flight](D:/%E5%AE%89%E8%A3%85%E8%BD%AF%E4%BB%B6/Dict/8.1.2.0/resultui/html/index.html#/javascript:;) [Height](D:/%E5%AE%89%E8%A3%85%E8%BD%AF%E4%BB%B6/Dict/8.1.2.0/resultui/html/index.html#/javascript:;)**  **(m)** | **Nozzle Voltage**  **(v)** | **Set Spray Concentration**  **(mL·acre^-1^)** | **The Angle of Solenoid Valve**  **(°)** | **Spray Time**  **（s）** | **Spray Area**  **（acre）** | **Test Group** | | | | **Control Group** | |
| --- | --- | --- | --- | --- | --- | --- | --- | --- | --- | --- | --- | --- | --- | --- |
|  |  |  |  |  |  |  |  |  | **Means of Spray Volume**  **(mL)** | **Actual Spray Concentration**  **(mL·acre^-1^)** | **Relative Deviation of Spray Concentration**  **(%)** | **Standard Deviation**  **(%)** | **Actual Spray Concentration**  **(mL·acre^-1^)** | **Relative Deviation of Spray Concentration**  **(%)** |
| **One-way Tests** | G1 | 3 | 4 | 8 | 4800 | 25.0 | 177.8 | 0.33 | **1733.18** | 5252.06 | **9.42** | **0.5728** | **5528.94** | **15.19** |
|  |  | 5 |  |  |  | 18.3 | 106.7 |  | **1597.05** | 4839.55 | **0.82** | **0.2466** | **5703.72** | **18.83** |
|  |  | 6 |  |  |  | 14.9 | 88.9 |  | **1516.72** | 4596.12 | **4.25** | **0.4579** | **3783.48** | **21.18** |
|  | G2 | 4 | 2 | 8 | 4800 | 25.5 | 188.4 |  | **1543.55** | 4677.42 | **2.55** | **0.491** | **6393.54** | **33.20** |
|  |  |  | 3 |  |  | 23.5 | 155.1 |  | **1672.68** | 5068.73 | **5.60** | **0.7802** | **5237.46** | **9.11** |
|  |  |  | 5 |  |  | 18.9 | 111.2 |  | **1645.50** | 4986.36 | **3.88** | **0.4253** | **6829.20** | **42.28** |
|  | G3 | 4 | 4 | 5 | 4800 | 18.9 | 111.2 |  | **1739.03** | 5269.79 | **9.79** | **1.5276** | **4298.64** | **10.45** |
|  |  |  |  | 6.5 |  | 20.3 | 121.2 |  | **1618.12** | 4903.39 | **2.15** | **0.6873** | **5302.5** | **10.47** |
|  |  |  |  | 10 |  | 23.0 | 148.2 |  | **1677.10** | 5082.12 | **5.88** | **0.4602** | **3516.84** | **26.73** |
|  | G4 | 4 | 4 | 8 | 3000 | 26.6 | 133.4 |  | **1062.57** | 3219.91 | **7.33** | **2.1081** | **5173.62** | **72.45** |
|  |  |  |  |  | 3900 | 24.1 |  |  | **1400.32** | 4243.39 | **8.80** | **0.907** | **4263.90** | **9.33** |
|  |  |  |  |  | 6000 | 18.3 |  |  | **1977.90** | 5993.64 | **0.11** | **0.0732** | **7272.18** | **21.20** |
| **Multivariate Tests** | T1 | 3 | 2 | 5 | 3600 | 28.6 | 209.7 |  | **1283.69** | 3889.97 | **8.05** | **2.1476** |  |  |
|  | T2 | 3 | 3 | 6.5 | 4200 | 27.1 | 197.6 |  | **1438.49** | 4359.06 | **3.79** | **0.7902** |  |  |
|  | T3 | 3 | 5 | 10 | 6000 | 21.2 | 161.7 |  | **1964.28** | 5952.36 | **0.79** | **0.4858** |  |  |
|  | T4 | 5 | 2 | 6.5 | 6000 | 18.8 | 137.5 |  | **2099.95** | 6363.48 | **6.06** | **1.3368** |  |  |
|  | T5 | 5 | 3 | 10 | 3600 | 24.7 | 130.1 |  | **1228.99** | 3724.21 | **3.45** | **2.0618** |  |  |
|  | T6 | 5 | 5 | 5 | 4200 | 14.5 | 76.2 |  | **1351.96** | 4096.85 | **2.46** | **0.8786** |  |  |
|  | T7 | 6 | 2 | 10 | 4200 | 23.8 | 139.8 |  | **1519.65** | 4605.00 | **9.64** | **1.1408** |  |  |
|  | T8 | 6 | 3 | 5 | 6000 | 10.0 | 88.9 |  | **1934.14** | 5861.03 | **2.32** | **0.6943** |  |  |
|  | T9 | 6 | 5 | 6.5 | 3600 | 2.5 | 68.4 |  | **1258.45** | 3813.48 | **5.93** | **0.7057** |  |  |

**Table 2**: The data of the system response time tests

| **[Group](file:///C:\\Users\\f\\Desktop\\plos%20one%20审稿意见及修改回复%20-%20副本.docx#/javascript:;)s** | **[Flight](file:///C:\\Users\\f\\Desktop\\plos%20one%20审稿意见及修改回复%20-%20副本.docx#/javascript:;) [Speed](file:///C:\\Users\\f\\Desktop\\plos%20one%20审稿意见及修改回复%20-%20副本.docx#/javascript:;)**  **(m·s^-1^)** | **[Flight](file:///C:\\Users\\f\\Desktop\\plos%20one%20审稿意见及修改回复%20-%20副本.docx#/javascript:;) [Height](file:///C:\\Users\\f\\Desktop\\plos%20one%20审稿意见及修改回复%20-%20副本.docx#/javascript:;)**  **(m)** | **Nozzle Voltage**  **(v)** | **The Set Spray**  **Concentration (mL·acre^-1^)** | **The Corresponding Time (s)** | **The Corresponding Distance (m)** |
| --- | --- | --- | --- | --- | --- | --- |
| A0 | 4 | 4 | 0 | 0 | / | / |
| A1 | 4 | 4 | 10 | 500 | 0.37 | 1.48 |
| A2 |  |  | 8 | 500 | 0.26 | 1.04 |
| A3 |  |  | 6.5 | 500 | 0.25 | 1.00 |
| A4 |  |  | 5 | 500 | **0.25** | **1.00** |
| A5 | 4 | 4 | 5 | 650 | 0.40 | 1.60 |
| A6 |  |  | 5 | 800 | 0.27 | 1.08 |
| A7 |  |  | 5 | 1000 | **0.47** | **1.88** |

The information of the means of spray volume, standard deviations, the values used to build graphs, the points extracted from images for analysis and other measures reported were in the table1 and table2. And we obtained actual spray concentration based on the means, we built graphs through the relative deviation of spray concentration and the standard deviation. The points extracted from images for analysis were track.
